# Frequency of severe reactions following penicillin drug provocation tests: A Bayesian meta‐analysis

**DOI:** 10.1002/clt2.12008

**Published:** 2021-06-21

**Authors:** António Cardoso‐Fernandes, Kimberly G. Blumenthal, Anca Mirela Chiriac, Isabel Tarrio, David Afonso‐João, Luís Delgado, João Almeida Fonseca, Luís Filipe Azevedo, Bernardo Sousa‐Pinto

**Affiliations:** ^1^ Department of Community Medicine, Information and Health Decision Sciences (MEDCIDS) Faculty of Medicine, University of Porto Porto Portugal; ^2^ Center for Health Technology and Services Research (CINTESIS) Faculty of Medicine, University of Porto Porto Portugal; ^3^ Division of Rheumatology, Allergy, and Immunology, Department of Medicine Massachusetts General Hospital Boston Massachusetts USA; ^4^ Harvard Medical School Harvard University Boston Massachusetts USA; ^5^ Department of Pulmonology Division of Allergy, Hôpital Arnaud de Villeneuve University Hospital of Montpellier Montpellier France; ^6^ UMR‐S 1136 INSERM‐Sorbonne Université Equipe Epidémiologie des Maladies Allergiques et Respiratoires (EPAR) Institut Pierre Louis d’Epidémiologie et de Santé Publique Paris France; ^7^ Basic and Clinical Immunology Unit, Department of Pathology, Faculty of Medicine University of Porto Porto Portugal

**Keywords:** adverse drug reactions, anaphylaxis, Bayesian meta‐analysis, drug allergy, drug challenge, drug provocation test, penicillin allergy, systematic review

## Abstract

**Background:**

Patients with a penicillin allergy label tend to have worse clinical outcomes and increased healthcare use. Drug provocation tests (DPT) are the gold‐standard in the diagnostic workup of penicillin allergy, but safety concerns may hinder their performance. We aimed to assess the frequency of severe reactions following a DPT in patients with reported allergy to penicillins or other *β*‐lactams.

**Methods:**

We performed a systematic review, searching MEDLINE, Scopus, and Web of Science. We included primary studies assessing participants with a penicillin allergy label who underwent a DPT. We performed a Bayesian meta‐analysis to estimate the pooled frequency of severe reactions to penicillin DPTs. Sources of heterogeneity were explored by subgroup and metaregression analyses.

**Results:**

We included 112 primary studies which included a total of 26,595 participants. The pooled frequency of severe reactions was estimated at 0.06% (95% credible interval [95% CrI] = 0.01%–0.13%; *I* ^2^ = 57.9%). Most severe reactions (80/93; 86.0%) consisted of anaphylaxis. Compared to studies where the index reaction was immediate, we observed a lower frequency of severe reactions for studies assessing non‐immediate index reactions (OR = 0.05; 95% CrI = 0‐0.31). Patients reporting anaphylaxis as their index reaction were found to be at increased risk of developing severe reactions (OR = 13.5; 95% CrI = 7.7–21.5; *I* ^2^ = 0.3%). Performance of direct DPTs in low‐risk patients or testing with the suspected culprit drug were not associated with clinically relevant increased risk of severe reactions.

**Conclusions:**

In patients with a penicillin allergy label, severe reactions resulting from DPTs are rare. Therefore, except for patients with potentially life‐threatening index reactions or patients with positive skin tests—who were mostly not assessed in this analysis ‐, the safety of DPTs supports their performance in the diagnostic assessment of penicillin allergy.

## 1 BACKGROUND

*β*‐Lactam antibiotics constitute the preferred treatment for many infections, but they are not typically prescribed to patients who report a past history of allergic reactions to this drug class.^1^ In fact, penicillins correspond to the drug class most patients report to be allergic—between 5% and 10% of individuals from the general population report having a penicillin allergy, and this frequency can reach up to 16% in hospitalized patients.^2^, ^3^, ^4^, ^5^, ^6^, ^7^ However, only a small fraction of these individuals (estimated in 2%–10% in the United States and 18%‐30% in Europe) have a true allergy to *β*‐lactams.^2^, ^7^, ^8^

Patients mislabeled as having a penicillin allergy more frequently receive antibiotics with a broader spectrum, often with lower efficacy and increased side‐effects, leading to poorer clinical outcomes, longer hospitalizations, higher risk of drug‐resistant and healthcare‐associated infections, and increased healthcare costs.^1^, ^2^, ^9^, ^10^, ^11^ As a result, evaluating and delabeling patients with penicillin allergy has both clinical and economic advantages.^12^

The diagnostic workup of a suspected penicillin allergy comprises a sequence of steps, typically including a complete clinical history, followed by skin tests and potentially in vitro tests (e.g., specific IgE quantification). Ultimately, if negative results are obtained with those tests, a drug provocation test (DPT; i.e., “drug challenge”), consisting in the controlled administration of a drug under strict clinical supervision, is considered to establish or rule out the diagnosis of penicillin allergy.^7^, ^13^, ^14^, ^15^, ^16^ In patients whose clinical history is poorly compatible with a true penicillin allergy, some experts advocate the performance of direct DPT (i.e., DPT without preceding in vivo or in vitro testing).^1^ On the contrary, in patients with history of potentially life‐threatening index reactions (e.g., Stevens–Johnson syndrome [SJS]/toxic epidermal necrolysis [TEN], severe anaphylaxis, or some severe specific organ manifestations), DPT are contraindicated.^16^

While DPTs are the gold‐standard in the diagnosis of penicillin allergy, the possibility of precipitating severe hypersensitivity reactions may prompt safety concerns.^16^ However, the frequency of such severe reactions has not been systematically evaluated. Therefore, in this systematic review and meta‐analysis, we aimed to quantify the frequency of severe hypersensitivity reactions following a DPT in patients reporting a penicillin (or *β*‐lactam) allergy, as well as to explore the impact of different patients' and methodological characteristics on the frequency of such severe reactions.

## 2 METHODS

This systematic review with meta‐analysis follows Preferred Reporting Items for Systematic Reviews and Meta‐Analyses guidelines and the recommendations of the Cochrane Handbook for Systematic Reviews.^17^, ^18^

### 2.1 Eligibility criteria

We included original studies reporting the frequency of severe reactions subsequent to DPTs in patients reporting a penicillin or *β*‐lactam allergy. Severe reactions were defined as episodes of anaphylaxis, shock, SJS/TEN, acute generalized exanthematous pustulosis, drug reaction with eosinophilia and systemic symptoms, acute interstitial nephritis, hemolytic anemia, serum sickness, drug fever, or other reactions described by the authors as severe and/or—if no additional information was provided—whose reaction treatment required more than antihistamines or corticosteroids (e.g., epinephrine) to subside. Other positive reactions to DPT were not considered severe, and therefore not taken into account.

We excluded studies deliberately performing DPTs with drugs from another antibiotic class, assessing allergy to cephalosporins exclusively or patients with specific diseases or occupations (e.g., only patients with cancer), or adopting a case‐control approach (as data from those studies do not permit calculation of the risk of severe reactions).

### 2.2 Information sources and search methods

We searched three electronic bibliographic databases (MEDLINE, Web of Science, and Scopus), through June 2019. Search queries are detailed in Table A1. References of included studies and of other relevant studies were further reviewed. No restriction on publication languages or dates were applied.

### 2.3 Study selection and data collection process

After duplicates removal, each study was independently assessed by two reviewers (researchers B.S.P. and A.C.F.), first by title and abstract screening, and then by full text reading. Data were independently extracted by two reviewers using a predefined online form purposely built for this study (a pilot version was built to assess the first 15 studies, and subsequently modified accordingly). For each study, we retrieved information on (i) the year of publication; (ii) country; (iii) participants' age group; (iv) setting (i.e., outpatients, inpatients or other); (v) timing of the index reaction (immediate reactions were defined as those occurring during the first hour after exposure to the culprit drug, and the remainder were classified as nonimmediate reactions^14^, ^15^); (vi) culprit drug class (i.e., whether studies included participants reporting an allergy to any *β*‐lactam or specifically to penicillins); (vii) whether penicillin re‐exposure occurred as part of a diagnostic workup or for therapeutic reasons; (viii) whether single dose, graded or prolonged (>24 h) DPTs were performed; (ix) the route of drug administration; (x) whether DPTs were preceded by skin/in vitro tests or directly performed; (xi) the drugs tested; and (xii) the period during which patients were followed for adverse reactions. In addition, for each primary study, we retrieved information on the number of participants undergoing a DPT, as well as on the number and type of subsequent severe reactions. Whenever provided, we separately retrieved these data for patients who reported immediate index reactions and for patients reporting anaphylaxis as their index reaction (we were not able to perform separate analyses for index reactions as the information necessary was not consistently provided on primary studies). Specific data regarding DPTs to penicillins were always preferred over data regarding DPTs to overall *β*‐lactam antibiotics. Disagreements between reviewers in study selection or data extraction were solved by consensus.

Full texts were carefully examined so as not to include the same results/patients more than once. Authors were contacted whenever full texts were not available (or in the two cases they were only available in a language authors were not fluent, with two received responses) or to provide relevant missing information.

### 2.4 Quality assessment

The quality of primary studies was independently assessed by two researchers using an adaptation of a tool developed for prevalence studies.^19^ Of the 11 items described, we used six items that were adequate for the aim of this study, namely: (i) if the study's target population was representative of the national population in relation to relevant variables; (ii) if the sample frame was representative of the target population; (iii) if some form of random or consecutive selection was used to select the sample; (iv) if the likelihood of nonresponse bias was minimal (defined as less than 25% follow‐up losses and/or participants with negative skin/in vitro tests not undergoing DPT); (v) if an acceptable/sufficiently complete definition of “severe reaction” was used in the study (or if allergic reactions were described in detail); and (vi) if the same methods of assessment and data collection were used for all subjects.

### 2.5 Quantitative synthesis of results

In order to quantitatively synthesize the frequency of severe reactions subsequent to DPTs, we performed Bayesian meta‐analyses following a random effects model based on a binomial likelihood (as described by Welton et al.^20^). We opted for this approach due to the large quantity of studies in which no severe reactions were observed. In fact, one of the advantages of a Bayesian meta‐analysis based on a binomial likelihood concerns its use of exact methods, dealing more adequately with proportions equal to zero (by contrast, a frequentist approach would imply the need for a continuity correction at least to the proportions equal to zero).^20^

Bayesian methods provide estimations of posterior probability distributions of the parameters of interest, based on prior probability distributions and on the observed data. In this study, based on the frequencies of severe reactions reported in primary studies, we obtained, through meta‐analytic methods of weighting, a probability distribution of the frequency of severe reactions. In addition, we obtained probability distributions for the odds ratio (OR) assessing the association between reporting anaphylaxis as index reaction and occurrence of severe reactions following a DPT. Of these posterior probabilities, we collected information on the mean values and respective 95% credible intervals (95% CrI; range of values within which, with 95% probability, the true frequency of severe reactions lies).^20^

We assessed heterogeneity—defined as the existence of differences beyond those that would be expected just by random sampling—by computing estimates of the *I* ^2^ statistic. An *I* ^2^ > 50% was indicative of substantial heterogeneity. Heterogeneity sources were explored by means of metaregression and subgroup analyses (i.e., a specific type of sensitivity analysis, consisting of separate meta‐analyses restricted to specific categories of retrieved variables). Exponentials of the metaregression coefficients were interpreted as OR.

Both for the effect size measure and for the τ parameter we used uninformative prior distributions (dnorm(0, 0.00001) and dgamma(0.00001, 0.00001), respectively). For each analysis, we ran at least 40,000 iterations with a burn‐in of 15,000 sample iterations. Meta‐analysis was performed using rjags package of software R (version 3.5.0).

## 3 RESULTS

### 3.1 Study selection

**FIGURE 1:**
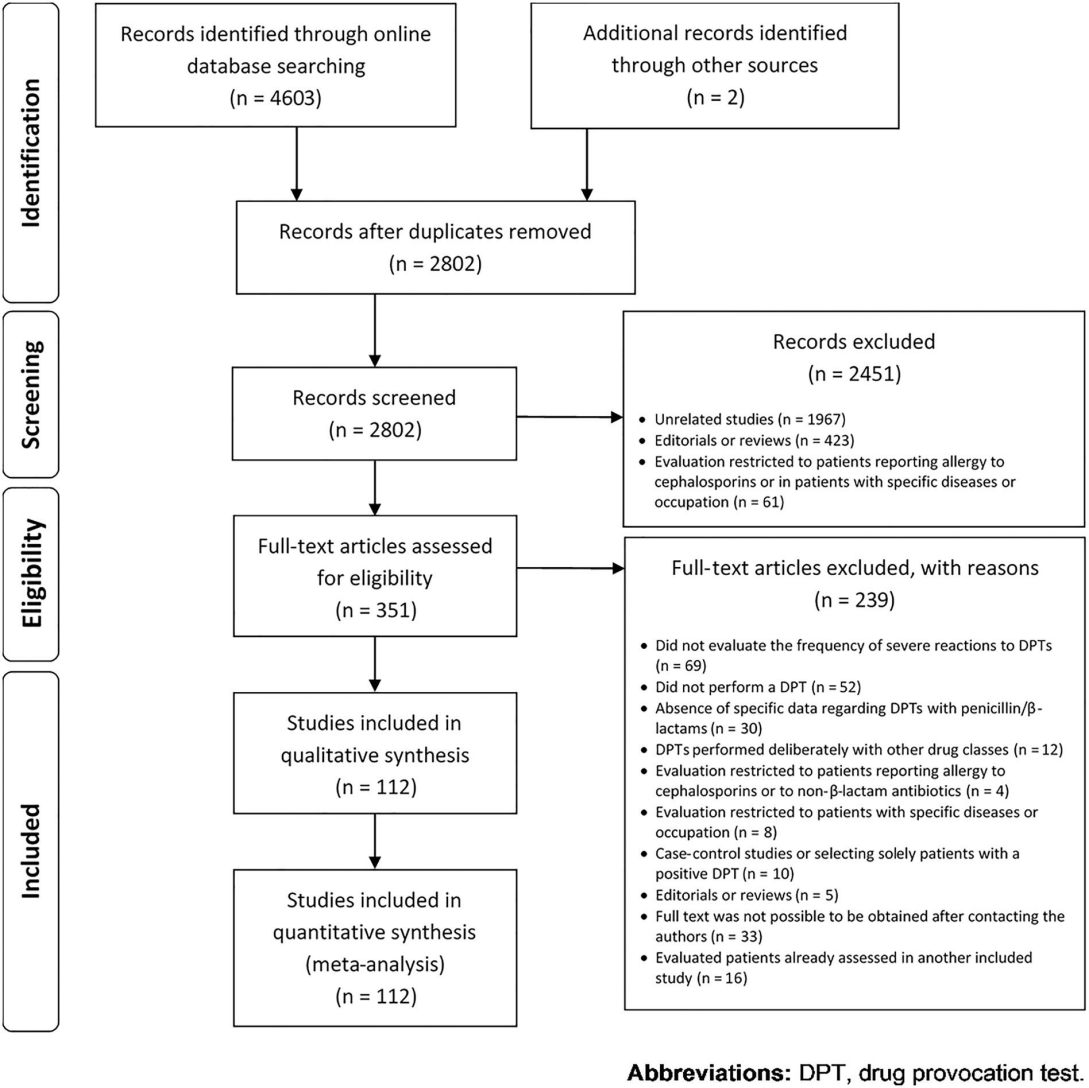
Flow diagram of study selection

With our search, we obtained 4603 records, of which 1803 were duplicates (Figure 1). After excluding 2451 records in the screening phase, 351 articles were fully read, of which a total of 112 studies were included in the systematic review.^21^, ^22^, ^23^, ^24^, ^25^, ^26^, ^27^, ^28^, ^29^, ^30^, ^31^, ^32^, ^33^, ^34^, ^35^, ^36^, ^37^, ^38^, ^39^, ^40^, ^41^, ^42^, ^43^, ^44^, ^45^, ^46^, ^47^, ^48^, ^49^, ^50^ ^1^, ^51^, ^52^, ^53^, ^54^, ^55^, ^56^, ^57^, ^58^, ^59^, ^60^, ^61^, ^62^, ^63^, ^64^, ^65^, ^66^, ^67^, ^68^, ^69^, ^70^, ^71^, ^72^, ^73^, ^74^, ^75^, ^76^, ^77^, ^78^, ^79^, ^80^, ^81^, ^82^, ^83^, ^84^, ^85^, ^86^, ^87^, ^88^, ^89^, ^90^, ^91^, ^92^, ^93^ ^101^, ^102^, ^103^, ^104^, ^105^, ^106^, ^107^, ^108^, ^109^, ^110^, ^111^, ^112^, ^113^, ^114^, ^115^, ^116^, ^117^, ^118^, ^119^, ^120^, ^121^, ^122^, ^123^, ^124^, ^125^, ^126^, ^127^, ^128^, ^129^, ^130^, ^131^, ^132^ Of these studies, 108 were included in all general analyses,^21^, ^22^, ^23^, ^24^, ^25^, ^26^, ^27^, ^28^, ^29^, ^30^, ^31^, ^32^, ^33^, ^34^, ^35^, ^36^, ^37^, ^38^ ^40^, ^41^, ^42^, ^43^, ^44^, ^45^, ^46^, ^47^, ^48^, ^49^, ^50^, ^51^, ^52^, ^53^, ^54^ ^1^, ^56^, ^57^, ^58^, ^59^, ^60^, ^61^, ^62^, ^63^, ^64^, ^65^, ^66^, ^67^, ^68^, ^69^, ^70^, ^71^, ^72^, ^73^, ^74^, ^75^, ^76^, ^77^, ^78^, ^79^, ^80^, ^81^, ^82^, ^83^, ^84^, ^85^, ^86^, ^87^, ^88^, ^89^, ^90^, ^91^, ^92^, ^93^, ^94^, ^95^, ^96^, ^97^, ^98^ ^101^, ^102^, ^103^, ^104^, ^105^, ^106^, ^107^, ^108^, ^109^, ^110^, ^111^, ^112^, ^113^, ^114^, ^115^, ^116^, ^117^ ^119^, ^120^, ^121^, ^122^, ^123^, ^124^, ^125^, ^126^, ^127^, ^128^, ^129^, ^130^, ^131^, ^132^ while the remaining four were only included in subgroup analyses as their participants partially overlapped with those of other included primary studies.^39^, ^55^, ^100^, ^118^ Sixteen studies found to be eligible were not included in this systematic review since they evaluated patients partially or fully assessed in another study, and which were not restricted to any particular characteristic that would render them available to be included in subgroup analyses.

### 3.2 Study characteristics

A summary of the included studies is presented in Table A2. Included studies were published between 1965 and 2019, and were mostly performed in North America (*n* = 46, 41.1%)^1^, ^21^, ^22^, ^23^, ^25^, ^26^, ^27^, ^31^, ^32^, ^34^, ^36^, ^37^, ^38^, ^40^, ^41^, ^44^, ^45^, ^46^, ^53^, ^58^, ^69^, ^70^, ^71^, ^74^, ^75^, ^84^, ^85^, ^88^, ^90^, ^96^, ^99^, ^102^, ^104^, ^105^, ^106^, ^107^, ^110^, ^111^, ^112^, ^114^, ^116^, ^122^ and Europe (*n* = 44, 39.3%).^1^, ^24^, ^28^, ^29^, ^30^, ^33^, ^35^, ^39^, ^42^, ^43^, ^47^, ^50^, ^52^, ^55^, ^56^, ^57^, ^59^, ^61^, ^62^, ^63^, ^64^, ^65^, ^66^, ^68^, ^73^, ^76^, ^77^, ^78^, ^79^, ^80^, ^82^, ^83^, ^86^, ^91^, ^92^, ^94^, ^97^, ^100^, ^109^, ^118^, ^121^, ^124^, ^128^ Thirty‐eight studies (34.9%) analyzed exclusively children^23^, ^28^, ^35^, ^36^, ^40^, ^51^, ^56^, ^58^, ^59^, ^60^, ^61^, ^63^, ^67^, ^73^, ^74^, ^77^, ^78^, ^82^, ^83^, ^86^, ^87^, ^89^, ^90^, ^95^, ^100^, ^103^, ^106^, ^107^, ^108^, ^109^, ^110^, ^113^, ^115^, ^118^, ^120^, ^121^, ^130^, ^132^ and 28 (25.7%) only analyzed adult patients.^21^, ^31^, ^32^, ^37^, ^38^, ^41^, ^49^, ^52^, ^53^, ^62^, ^68^, ^71^, ^76^, ^79^, ^88^, ^94^, ^101^, ^102^, ^104^, ^105^, ^114^, ^117^, ^123^, ^124^, ^125^, ^127^, ^128^, ^131^ Most studies only included outpatients (*n* = 69, 66.3%).^28^, ^29^, ^30^, ^32^, ^33^, ^34^, ^36^, ^39^, ^40^, ^42^, ^43^, ^44^, ^45^, ^47^, ^48^, ^49^, ^50^ ^52^, ^53^, ^54^, ^55^, ^57^, ^59^, ^60^, ^61^, ^62^, ^63^, ^64^, ^65^, ^66^, ^68^, ^72^ ^73^, ^75^, ^76^, ^78^, ^79^, ^80^, ^81^, ^84^, ^85^, ^86^, ^90^, ^91^, ^92^, ^93^, ^95^, ^97^, ^98^, ^99^, ^100^ ^103^, ^104^, ^107^, ^108^, ^110^, ^111^, ^115^, ^118^ ^120^, ^121^, ^122^, ^124^, ^125^, ^126^, ^128^, ^129^, ^130^, ^132^ From the 96 studies reporting information on the timing of the index reaction, 15 studies (15.6%) only evaluated patients reporting immediate allergic reactions,^24^, ^30^, ^33^, ^38^, ^39^, ^40^, ^42^, ^54^, ^60^ ^67^, ^80^, ^91^, ^102^, ^103^, ^130^ and 12 studies (10.7%) exclusively evaluated patients reporting non‐immediate index reactions.^50^, ^55^, ^56^, ^76^, ^78^, ^79^, ^86^, ^95^, ^98^, ^100^, ^119^, ^121^

Prolonged challenges were performed in 41 (39.4%) of the included studies,^22^, ^26^, ^28^, ^29^, ^31^, ^36^, ^37^, ^38^, ^41^, ^45^, ^47^, ^48^, ^49^, ^50^ ^55^, ^56^, ^61^, ^65^, ^68^, ^77^, ^78^, ^80^, ^82^, ^86^, ^89^, ^95^, ^98^, ^100^ ^101^, ^108^, ^110^, ^115^, ^118^, ^119^, ^120^, ^121^, ^124^, ^128^, ^129^, ^130^, ^132^ while graded challenges were performed in 37 (35.6%) studies,^21^, ^23^, ^24^, ^27^, ^33^, ^35^, ^39^, ^42^, ^51^, ^52^, ^54^, ^57^, ^58^, ^59^, ^60^, ^62^, ^63^, ^67^, ^69^, ^72^, ^73^, ^75^, ^76^, ^87^, ^88^, ^90^, ^91^, ^94^, ^97^, ^99^, ^103^, ^106^, ^107^, ^116^, ^122^, ^126^, ^127^ and 17 (16.3%) studies opted for single dose DPTs.^30^, ^34^, ^40^, ^44^, ^53^, ^70^, ^71^, ^79^, ^84^, ^96^, ^104^, ^105^, ^111^, ^112^, ^117^, ^125^, ^131^ Half of the studies (*n* = 59, 52.7%) performed a DPT with the suspected culprit drug.^25^, ^33^, ^35^, ^36^, ^39^, ^40^, ^47^, ^48^, ^49^, ^50^, ^51^, ^54^, ^55^, ^56^, ^57^ ^59^, ^60^, ^61^, ^65^, ^66^, ^67^, ^68^, ^69^, ^71^, ^73^, ^75^, ^76^, ^77^, ^78^, ^80^, ^81^, ^83^, ^84^, ^85^, ^86^, ^87^, ^89^, ^90^, ^91^, ^92^, ^93^, ^94^, ^95^, ^98^, ^100^ ^103^, ^108^, ^109^, ^110^, ^115^, ^117^, ^118^, ^121^, ^124^, ^128^, ^129^, ^130^, ^132^ In 12 studies (10.9%), direct DPTs (DPTs without previous skin/in vitro tests) were performed,^69^, ^82^, ^87^, ^90^, ^95^, ^110^, ^119^, ^121^, ^122^, ^125^, ^127^, ^128^ while in 87 studies (79.1%) challenges were always preceded by previous tests.^21^, ^22^, ^23^, ^24^, ^26^, ^27^, ^28^, ^29^, ^30^, ^31^, ^32^, ^33^, ^34^, ^35^, ^36^, ^37^, ^38^, ^39^, ^40^, ^41^, ^42^, ^44^, ^45^, ^46^, ^47^, ^48^, ^49^, ^50^, ^51^, ^52^, ^53^, ^54^, ^55^, ^56^, ^57^, ^58^, ^60^, ^61^, ^62^, ^63^, ^64^, ^65^, ^66^, ^67^, ^68^, ^70^, ^71^, ^72^, ^73^, ^74^, ^75^, ^76^, ^77^, ^78^, ^79^, ^80^, ^81^, ^83^, ^84^, ^86^, ^88^, ^89^, ^91^, ^92^, ^93^, ^94^, ^96^, ^97^, ^98^, ^100^, ^101^, ^102^, ^103^, ^104^, ^106^, ^107^, ^108^, ^109^, ^114^, ^118^, ^120^, ^123^, ^124^, ^129^, ^130^, ^131^, ^132^ Eleven studies (10.0%) included patients undergoing both direct DPTs and DPTs preceded by other tests.^37^, ^59^, ^85^, ^99^, ^105^, ^111^, ^112^, ^115^, ^116^, ^117^, ^126^

### 3.3 Frequency of severe reactions

In the included studies, a total of 26,595 participants underwent a DPT, of whom 93 experienced severe reactions (0.4%). Most reactions were classified as anaphylaxis (*n* = 80; 86.0%), followed by serum sickness (*n* = 7; 7.5%), and maculopapular exanthema with systemic symptoms (*n* = 6; 6.5%). No fatal reactions were observed (Table 1).

**TABLE 1:** Outcomes of drug provocation tests (DPT) across the included primary studies

	*N* (meta‐analytical frequency; 95% CrI; *I* ^2^)
Patients who performed a DPT	26,595
Patients with a positive DPT	1300 (3.8%; 2.9%–4.7%; 19.8%)
Patients with severe hypersensitivity reactions	93 (0.06%; 0.01%–0.13%; 57.9%)
Anaphylaxis^a^	80 (0.03%; 0%–0.08%; 65.7%)
Serum sickness	7 (0.01%; 0%–0.03%; 22.7%)^b^
SJS/TEN	0
AGEP/DRESS	0
Acute interstitial nephritis	0
Hemolytic anemia	0
Drug fever	0
Others^c^	6 (0.001%; 0%–0.01%; 68.4%)^b^
Patients with fatal hypersensitivity reactions	0

Abbreviations: AGEP, acute generalized exanthematous pustulosis; CrI, credible interval; DRESS, drug reaction with eosinophilia and systemic symptoms; SJS, Stevens–Johnson Syndrome; TEN, toxic epidermal necrolysis.

^a^ Includes anaphylactic reactions described as such in included primary studies, as well as reactions described in those studies and which are compatible with anaphylaxis criteria.

^b^ Meta‐analytical frequency of severe nonanaphylactic reactions = 0.02% (95% CrI = 0%–0.04%; *I* ^2^ = 44.2%).

^c^ Maculopapular exanthemas with systemic symptoms not qualifying as DRESS.

The Bayesian meta‐analysis identified a frequency of severe reactions of 0.06% (95% CrI = 0.01%–0.13%), albeit with severe heterogeneity (*I* ^2^ = 57.9%; Table 2). The meta‐analytical frequency of anaphylaxis was of 0.03% (95% CrI = 0%–0.08%; *I* ^2^ = 65.7%), while it was of 0.02% (95% CrI = 0%–0.04%; *I* ^2^ = 44.2%) for nonanaphylactic severe reactions. The results of univariable metaregression and subgroup analyses are presented in Tables 2 and 3. A clear decrease in the risk of severe reactions was observed for studies assessing patients reporting non‐immediate reactions (OR = 0.05; 95% CrI = 0–0.31), for studies performing single‐dose DPTs (OR = 0.08; 95% CrI = 0–0.47), and for studies performed in North America (OR = 0.25; 95% CrI = 0.04–0.80; Table A3 presents results stratified by region). We did not find a clear increase in the risk of severe reactions—considering the results of both metaregression and subgroup analysis—with DPTs performed with the suspected drug (OR = 1.74; 95% CrI = 0.27–5.89), with the performance of direct DPTs (OR = 1.00; 95% CrI = 0.47–4.16), or with the possibility of reporting reactions for more than one day (OR = 1.54; 95% CrI = 0.06–8.34).

**TABLE 2:** Results of metaregression and subgroup analyses for the frequency of severe reactions following penicillins drug challenges

	Number of studies	Number of patients	Subgroup analyses		Univariable metaregression—OR (95% CrI) [% iterations with OR > 1]
			Percent of severe reactions (95% CrI)	*I* ^2^	
All	108	26,595	0.06 (0.01–0.13)	57.9 %	
Year of publication	108	26,595	^a^	^a^	1.00 (0.99–1.01) [13%]
Geographic region					
Europe	40	14,049	0.10 (0.01–0.26)	61.8 %	3.29 (0.62–10.33) [81%]
North America	46	10,198	0.08 (0–0.17)	16.9 %	0.25 (0.04–0.80) [1%]
Age group					
Children	37	8223	0.14 (0.02–0.32)	44.6 %	^b^
Adults^c^	28	3787	0.21 (0.02–0.37)	15.5 %	1.16 (0.16–3.83) [34%]
Setting					
Outpatients only	65	18,748	0.07 (0.01–0.17)	59.5 %	^b^
Inpatients only	17	1732	0.03 (0–0.09)	20.2 %	0.29 (0.01–1.73) [6%]
Type of clinic where patients were tested					
Nonallergy clinic	24	4206	0.02 (0–0.04)	55.4 %	^b^
Allergy clinic	68	18,800	0.06 (0.01–0.15)	59.7 %	12.07 (0.43–94.01) [13%]
Timing of index reaction					
Immediate	24	3162	0.29 (0.01–1.00)	64.4 %	^b^
Nonimmediate	22	4271	0.001 (0–0.001)	97.0 %	0.05 (0–0.31) [0%]
Culprit drug class (index reaction)					
Any penicillin	71	11,658	0.02 (0–0.08)	71.0 %	^b^
Any *β*‐lactam	37	14,937	0.14 (0.03–0.29)	45.7 %	3.30 (0.50–12.89) [83%]
Context of DPT/drug exposure					
Diagnostic DPT	96	24,044	0.06 (0.01–0.13)	60.4 %	^b^
Therapeutic exposure	10	2086	0.14 (0.09–0.18)	0.5 %	1.22 (0.03–6.63) [31%]
DPT dosing					
Single	17	4893	0.01 (0–0.04)	6.6 %	0.08 (0–0.47) [0%]
Graded^d^	36	7948	0.07 (0–0.25)	67.0 %	4.32 (0.39–18.81) [86%]
Prolonged^d^	38	9085	0.07 (0–0.20)	59.5 %	2.50 (0.24–10.65) [68%]
Route of drug administration					
Only oral	81	17,193	0.08 (0.02–0.17)	46.0 %	^b^
Oral and parenteral	18	8558	0.04 (0–0.21)	82.5 %	2.18 (0.24–8.90) [67%]
Diagnostic workup					
DPT preceded by previous tests	96	23,265	0.07 (0.02–0.15)	53.7 %	^b^
Direct DPT	21	3804	0.02 (0–0.12)	74.8 %	1.00 (0.07–4.16) [33%]
Drugs tested in the DPT					
Suspected drug not tested	44	9298	0.05 (0–0.17)	59.5 %	^b^
Suspected drug tested	55	14,447	0.06 (0.01–0.15)	62.1 %	1.74 (0.27–5.89) [60%]
Number of drugs tested in the DPT					
One drug	90	21,797	0.07 (0.02–0.16)	47.9 %	^b^
More than one drug	11	3565	0.90 (0–1.20)	83.8 %	7.17 (0.68–32.20) [93%]
Reporting time for severe reactions					
<1 Day	11	1430	0.20 (0.04–0.41)	2.5 %	^b^
≥1 Day	76	21,551	0.04 (0–0.10)	64.0 %	1.54 (0.06–8.34) [38%]
Studies methodological quality					
<3 Items classified as “high risk of bias”	90	24,569	0.06 (0.01–0.14)	59.2 %	^b^
≥3 Items classified as “high risk of bias”	18	2026	0.11 (0.01–0.26)	4.1 %	0.78 (0.03–3.66) [22%]

Abbreviations: CrI, credible interval; DPT, drug provocation test; OR, odds ratio.

^a^ No subgroup analysis performed, as this is a continuous variable (we were only able to perform metaregression analysis).

^b^ Reference category.

^c^ When analyzing together adults and young adults (≥15 years old), we obtained a frequency of severe reactions of 0.06% (0–0.24%; *I* ^2^ = 62.6%).

^d^ When comparing studies performing graded and prolonged DPTs, we obtained an OR = 1.17 (0.11–5.01), favoring prolonged DPTs.

**TABLE 3:** Results of subgroup analyses for the frequency of severe reactions following penicillin drug challenges in patients reporting index immediate reactions

	Number of studies	Number of patients	Subgroup analyses	
			Percent of severe reactions (95% CrI)	*I* ^2^
Children				
Index immediate reactions	10	545	0.64 (0–1.94)	25.3 %
Index immediate reactions with testing of the suspected culprit drug	9	526	0.82 (0.01–1.94)	23.5 %
Adults				
Index immediate reactions	4	199	1.62 (0.28–4.05)	4.5 %
Index immediate reactions with testing of the suspected culprit drug	2	95	^a^	^a^
Outpatients				
Index immediate reactions	16	2848	0.26 (0–1.16)	77.9 %
Index immediate reactions with testing of the suspected culprit drug	13	2612	0.80 (0–1.21)	77.1 %
Inpatients				
Index immediate reactions	2	64	^b^	^b^
Index immediate reactions with testing of the suspected culprit drug	1	2	^b^	^b^

Abbreviations: CrI, credible interval; DPT, drug provocation test; OR, odds ratio.

^a^ Convergence not reached;

^b^ No cases of severe reactions were observed in the studies assessing inpatients reporting index immediate reactions.

**TABLE 4:** Limitations of current evidence and key aspects for future studies

Limitations of current evidence	Key requirements needed for future studies
Heterogeneity and insufficient description of eligibility criteria	Clear description of eligibility criteria
Absence of studies with representative national or state‐wide representation	Conduction of multicentric studies with standardized methods
Inconsistent reporting of participants' demographic and clinical data	Clear description of the methodology used for performing DPT
Inconsistent or incomplete description of DPT procedures	Standardization of DPTs protocols
Potential selection bias related to the exclusion of less and/or more severe allergic patients	Consistent and detailed report of severe reactions to DPTs (e.g. clinical manifestations and timing)
Limited data from Latin America, Asia and Africa	Publication of anonymized patient‐level data OR presentation of stratified results by different reaction phenotypes and risk groups

Abbreviation: DPT, drug provocation test.

A total of 565 participants in 29 primary studies had reported anaphylaxis as their index reaction, of whom 32 experienced severe reactions. This corresponds to a meta‐analytical pooled frequency of 4.64% (95% CrI = 0.79%‐7.43%; *I* ^2^ = 11.4%) versus 0.09% (95% CrI = 0.01%–0.18%; *I* ^2^ = 42.0%) for the remaining participants. We, therefore, observed a strong association between reporting anaphylaxis as index reaction and occurrence of severe reactions following a DPT (OR = 13.48; 95% CrI = 7.68–21.53, *I* ^2^ = 0.3%).

### 3.4 Risk of bias of individual studies

**FIGURE 2:**
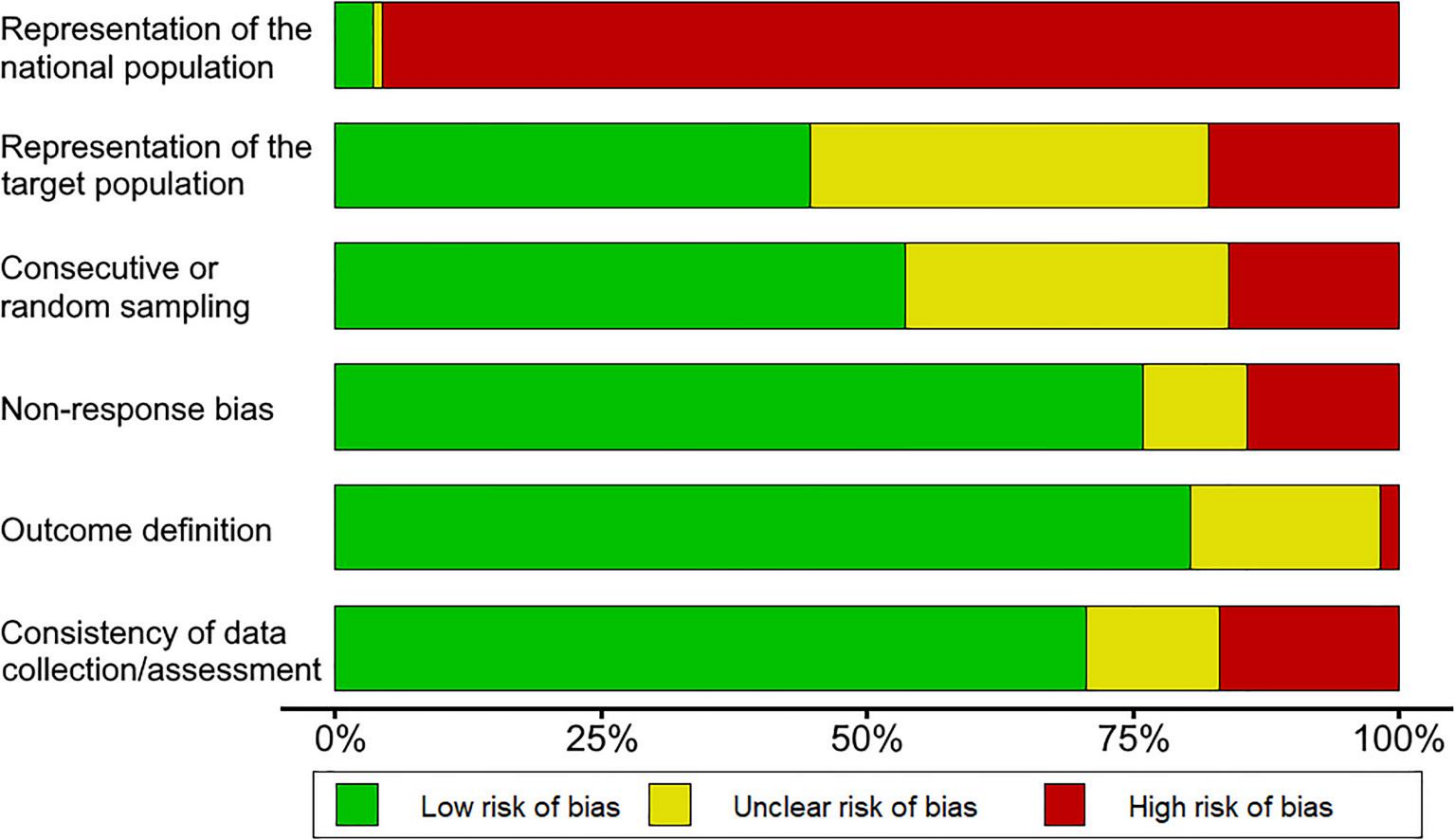
Risk of bias graph for included primary studies

A risk of bias graph is presented in Figure 2, and the complete analysis of the risk of bias of individual studies may be found in Table A4. Most studies had a high or unclear risk of bias in terms of sample representation. Nevertheless, most studies presented a low risk of bias regarding the other parameters evaluated. A similar frequency of severe reactions following DPTs was found in studies with three or more items classified as “high risk of bias” (0.11%, 95% CrI = 0.01%–0.26%; *I* ^2^ = 4.1%),^21^, ^25^, ^26^, ^32^, ^34^, ^37^, ^38^, ^41^, ^46^, ^49^, ^60^, ^96^, ^114^, ^117^, ^119^, ^123^, ^127^, ^128^ when compared with the remaining studies (0.06%, 95% CrI = 0.01%–0.14%; *I* ^2^ = 59.2%).^22^, ^23^, ^24^, ^27^, ^28^, ^29^, ^30^, ^31^, ^33^, ^35^, ^36^, ^40^, ^42^, ^43^, ^44^, ^45^, ^47^, ^48^, ^50^, ^51^, ^52^, ^53^, ^54^, ^56^, ^57^, ^58^, ^61^, ^62^, ^63^, ^64^, ^65^, ^66^, ^67^, ^68^, ^69^, ^70^, ^71^, ^72^, ^73^, ^74^, ^75^, ^76^, ^77^, ^78^, ^79^, ^80^, ^81^, ^82^, ^83^, ^84^, ^85^, ^86^, ^87^, ^88^, ^89^, ^90^, ^91^, ^92^, ^93^, ^94^, ^95^, ^97^, ^98^, ^99^, ^101^, ^102^, ^103^, ^104^, ^105^, ^106^, ^107^, ^108^, ^109^, ^110^, ^111^, ^112^, ^113^, ^115^, ^116^, ^120^, ^121^, ^122^, ^124^, ^125^, ^126^, ^129^, ^130^, ^131^, ^132^

## 4 DISCUSSION

This systematic review included 112 primary studies assessing the frequency of severe reactions following DPTs to penicillins. Results of the meta‐analysis suggest that severe reactions are rare, estimated at a frequency of 0.06%, corresponding to approximately an average of one severe reaction for each 1700 patients undergoing a DPT. Additionally, from the 26,595 assessed patients who underwent a DPT, none had a subsequent fatal reaction. However, it is worth noting that in studies in which direct challenges were not performed, more than 98% DPTs were performed in patients who had had negative skin or in vitro tests. In addition, most of the included studies, following international recommendations, did not challenge patients with a reported history of a severe or life‐threatening index reaction ‐ 79 studies explicitly reported severe cutaneous adverse reactions (e.g., SJS/TEN, which are contraindications for DPTs^7^, ^15^, ^16^) as exclusion criteria, of whom 30 did not test patients with history of anaphylaxis or anaphylactic shock either. In fact, when considering patients with an index reaction of anaphylaxis, the estimated frequency of severe reaction rises to 6.0%.

Our results should be carefully interpreted on account of the observed heterogeneity. Such heterogeneity indicates that across included studies, there are important differences in the frequency of severe reactions. To identify the variables explaining such differences, we performed metaregression and subgroup analyses, observing a lower frequency of severe reactions in studies evaluating patients reporting nonimmediate reactions. This mirrors the fact that nonimmediate reactions are typically mild, with the exception of SJS/TEN or other very rare potentially life‐threatening reactions (which are contraindications for DPTs).^98^ Furthermore, particularly in children, infectious and other nonallergic rashes are often erroneously reported as nonimmediate hypersensitivity reactions.^1^, ^7^, ^98^ On the other hand, we observed a lower frequency of severe reactions following single‐dose DPTs, which may be explained by selection biases (i.e., patients reporting less severe index reactions having greater chances of receiving single dose DPT), by the lower exposure of participants to the tested drugs, and by the fact that studies opting for single dose DPTs often observed the participants for a smaller period of time (possibly not registering later reactions). However, a similar frequency of severe reactions was observed when comparing studies performing graded versus prolonged DPTs. Finally, we did not observe a clear increase in the frequency of severe reactions when analyzing studies that performed DPTs with the suspected drug (as supported by international recommendations^16^). We did not observe either a higher frequency of severe reactions in those studies performing direct DPTs. However, care should be taken when interpreting those results as those studies mainly included patients deemed by allergists to have a low risk clinical history.

The region was also identified as a variable potentially explaining heterogeneity. In fact, in European studies, we observed higher frequency of severe hypersensitivity reactions and lower heterogeneity when compared to their North American counterparts, and a more evident increased risk of such reactions among adults. These differences may point to regional differences in the type of assessed patients, with a possibly higher predominance of low‐risk patients in North America.

This study has some limitations, in part due to the characteristics of primary studies. In particular, there is the possibility of selection biases, which may have resulted in overestimation or underestimation of the true frequency of severe reactions. In fact, 77% of primary studies explicitly reported to be performed in allergy clinics and/or involving allergy specialists, potentially affecting sample representativity. Also, during the selection process, 69 primary studies were excluded for not reporting the frequency of severe reactions—it is possible or potentially even likely that in those studies, severe reactions were not mentioned because they did not occur. This would render our 0.06% estimate an overestimate of the true frequency of severe reactions following a DPT. In addition, the exclusive assessment of patients referred to specialized clinics and/or lack of testing of patients with unconvincing penicillin allergy histories would also result in overestimating the frequency of severe reactions (for instance, we would expect that patients referred to specialized clinics may represent those with a higher risk of having a true penicillin allergy and that no testing would be performed in patients with intolerance reaction histories). On the other hand, in most studies, only patients with no history of particularly severe index reactions and with negative skin or in vitro tests underwent DPTs. This could, in turn, have resulted in an underestimation of the frequency of severe reactions.

Another important limitation concerns the severe heterogeneity found, mirroring not only the nature of this meta‐analysis (i.e., a quantitative synthesis of frequencies of a rare event), but also the differences in eligibility criteria and DPTs protocols across primary studies. In order to explore possible sources of heterogeneity, we performed metaregression and subgroup analyses, ceasing to detect severe heterogeneity in 11 out of 27 subgroup analyses. Unfortunately, on account of the scarcity of severe reactions, we were not able to perform multivariable metaregression analyses to identify adjusted moderators of heterogeneity. We attempted to circumvent this limitation by performing stratified analyses on the region.

Finally, information on index reactions was inconsistently reported across primary studies—except for anaphylaxis, we were not able to assess the risk of severe reactions associated with each type of index reaction.

The main strength of this study is its meta‐analytical approach for the quantitative synthesis of rare events. The main advantage of Bayesian meta‐analysis based on a binomial likelihood concerns its use of exact methods, allowing for dealing more adequately with zero‐cells (in this case corresponding to the majority of included primary studies, in which no severe reactions to DPTs were observed). By contrast, classical frequentist meta‐analytical methods would possibly result in an overestimation of the true frequency of such reactions.^133^ In addition, for the Bayesian meta‐analysis, we used noninformative priors, whose effect was further diluted by including a large number of primary studies, further decreasing the risk of priors dominating the results.^134^ Another methodologic strength concerns the performance of metaregression and subgroup analyses, aiming to identify patient or clinical characteristics associated with differences in the outcomes. Finally, we performed a comprehensive search, encompassing three different electronic bibliographic databases and not using exclusion criteria based on the date or language of publication.

In conclusion, and from a clinical point of view, this study suggests that overall, severe reactions are rare, occurring at an average of one reaction for each 1700 patients undergoing a DPT. In addition, the included primary studies did not report any fatal reactions; indeed, in a comprehensive search of the literature beyond the eligibility criteria of this systematic review, we only found one death described after a DPT to a penicillin, although this case was potentially attributable to resensitization to clavulanate.^135^ However, our results also point that the risk of a severe reaction is not the same for all patients. In fact, our overall results cannot be generalized to patients with potentially life‐threatening non‐IgE‐mediated index reactions (e.g., SJS/TEN)—in whom DPT is contraindicated—or to patients with positive penicillin skin tests, and we also identified that patients reporting an index anaphylactic reaction had almost 80 times more risk of developing severe reactions to DPT than the remaining participants. In fact, only one in each 100,000 DPT in patients reporting nonimmediate reactions are expected to result in severe reactions. This value rises to one in each 345 DPT in patients reporting immediate reactions, and to one in each 22 DPT in patients reporting a history of anaphylaxis. This highlights the importance of a thorough characterization of the index reaction (e.g., by a structured clinical history) prior to the diagnostic workup. Consistently, we observed that more than three‐quarters of reported severe reactions consisted of anaphylaxis, highlighting the need for prompt recognition and treatment of anaphylactic reactions. A detailed characterization of the index reaction may also identify those low‐risk patients who may undergo a direct DPT, in whom our results did not identify an increased risk of severe reactions.^136^, ^137^ Testing the suspected culprit drug did not associate with clear increased risk of severe reactions, and therefore should be encouraged.^138^

To the best of our knowledge, this is the first systematic review with meta‐analysis assessing the frequency of severe reactions following DPTs. Future primary studies may allow for a more thorough exploration of this issue, by providing more details on their methodology (particularly regarding eligibility criteria and DPT procedures) or even anonymized individual participant‐level data (Table 4). The results of this study support, from a safety point of view, the performance of DPTs during the diagnostic workup of penicillin allergy, particularly if a detailed allergy history has been obtained, evidence‐based recommendations are followed and there is appropriate supervision by an allergy specialist. This is particularly important, since delabeling patients reporting a penicillin allergy has been recommended as an antibiotic stewardship tool, to contribute to a more adequate prescription of antibiotics, minimizing patients' risks and improving clinical and economic outcomes.

## CONFLICT OF INTEREST

Kimberly G. Blumenthal has a clinical decision support tool for inpatient beta‐lactam allergy evaluation used at Partners HealthCare Systems and licensed to Persistent Systems.

## AUTHOR CONTRIBUTIONS

António Cardoso‐Fernandes: data curation (equal); formal analysis (equal); investigation (equal); methodology (equal); writing‐original draft (equal). Kimberly G. Blumenthal: conceptualization (equal); writing‐original draft (equal); writing‐review and editing (equal). Anca Mirela Chiriac: conceptualization (equal); writing‐original draft (equal); writing‐review and editing; (equal). Isabel Tarrio: data curation (equal); formal analysis (equal); investigation (equal). David Afonso‐João: formal analysis (equal); investigation (equal). Luis Delgado: conceptualization (equal); writing‐review and editing (equal). João Almeida Fonseca: conceptualization (equal); writing‐review and editing (equal). Luís Filipe Azevedo: conceptualization (equal); formal analysis (equal); methodology (equal); writing‐review and editing (equal). Bernardo Sousa‐Pinto: conceptualization (equal); data curation (equal); formal analysis (equal); investigation (equal); methodology (equal); writing‐original draft (equal).

## References

[1] Shenoy ES , Macy E , Rowe T , Blumenthal KG . Evaluation and management of penicillin allergy: a review. J Am Med Assoc. 2019;321(2):188‐199. 10.1001/jama.2018.19283. 30644987

[2] Blumenthal KG , Peter JG , Trubiano JA , Phillips EJ . Antibiotic allergy. Lancet. 2019;393(10167):183‐198. 10.1016/s0140-6736(18)32218-9. 30558872 PMC6563335

[3] Gomes E , Cardoso MF , Praça F , Gomes L , Mariño E , Demoly P . Self‐reported drug allergy in a general adult Portuguese population. Clin Exp Allergy. 2004;34(10):1597‐1601. 10.1111/j.1365-2222.2004.02070.x. 15479276

[4] Macy E , Poon K‐YT . Self‐reported antibiotic allergy incidence and prevalence: age and sex effects. Am J Med. 2009;122(8):778. 10.1016/j.amjmed.2009.01.034. 19635279

[5] Harandian F , Pham D , Ben‐Shoshan M . Positive penicillin allergy testing results: a systematic review and meta‐analysis of papers published from 2010 through 2015. Postgrad Med. 2016;128(6):557‐562. 10.1080/00325481.2016.1191319. 27240423

[6] Sousa‐Pinto B , Fonseca JA , Gomes ER . Frequency of self‐reported drug allergy: a systematic review and meta‐analysis with meta‐regression. Ann Allergy Asthma Immunol. 2017;119(4):362‐373. 10.1016/j.anai.2017.07.009. 28779998

[7] Joint Task Force on practice parameter, American Academy of allergy Asthma and Immunology, and American College of allergy Asthma and Immunology . Drug allergy: an updated practice parameter. Ann Allergy Asthma Immunol. 2010;105(4):259‐273. 10.1016/j.anai.2010.08.002. 20934625

[8] Solensky R . Allergy to β‐lactam antibiotics. J Allergy Clin Immunol. 2012;130(6):1442. 10.1016/j.jaci.2012.08.021. 23195529

[9] Sousa‐Pinto B , Cardoso‐Fernandes A , Araújo L , Fonseca JA , Freitas A , Delgado L . Clinical and economic burden of hospitalizations with registration of penicillin allergy. Ann Allergy Asthma Immunol. 2018;120(2):190‐194. 10.1016/j.anai.2017.11.022. 29413343

[10] Blumenthal KG , Lu N , Zhang Y , Li Y , Walensky RP , Choi HK . Risk of meticillin resistant Staphylococcus aureus and Clostridium difficile in patients with a documented penicillin allergy: population based matched cohort study. BMJ. 2018;361:k2400. 10.1136/bmj.k2400. 29950489 PMC6019853

[11] Blumenthal KG , Ryan EE , Li Y , Lee H , Kuhlen JL , Shenoy ES . The impact of a reported penicillin allergy on surgical site infection risk. Clin Infect Dis. 2018;66(3):329‐336. 10.1093/cid/cix794. 29361015 PMC5850334

[12] Sousa‐Pinto B , Blumenthal KG , Macy E , et al. Penicillin allergy testing is cost‐saving: an economic evaluation study. Clin Infect Dis. 2020. 10.1093/cid/ciaa194. PMC7958749 32107530

[13] Blanca M , Romano A , Torres MJ , et al. Update on the evaluation of hypersensitivity reactions to betalactams. Allergy. 2009;64(2):183‐193. 10.1111/j.1398-9995.2008.01924.x. 19133923

[14] Romano A , Blanca M , Torres MJ , et al. Diagnosis of nonimmediate reactions to β‐lactam antibiotics. Allergy. 2004;59(11):1153‐1160. 10.1111/j.1398-9995.2004.00678.x. 15461594

[15] Torres MJ , Blanca M , Fernandez J , et al. Diagnosis of immediate allergic reactions to beta‐lactam antibiotics. Allergy. 2003;58(10):961‐972. 10.1034/j.1398-9995.2003.00280.x. 14510712

[16] Aberer W , Bircher A , Romano A , et al. Drug provocation testing in the diagnosis of drug hypersensitivity reactions: general considerations. Allergy. 2003;58(9):854‐863. 10.1034/j.1398-9995.2003.00279.x. 12911412

[17] Liberati A , Altman DG , Tetzlaff J , et al. The PRISMA statement for reporting systematic reviews and meta‐analyses of studies that evaluate healthcare interventions: explanation and elaboration. BMJ. 2009;339:b2700. 10.1136/bmj.b2700. 19622552 PMC2714672

[18] Higgins JPT , Thomas J , Chandler J , et al. (eds.). Cochrane Handbook for Systematic Reviews of Interventions version 6.0 (updated July 2019): Cochrane; 2019. www.training.cochrane.org/handbook.

[19] Hoy D , Brooks P , Woolf A , et al. Assessing risk of bias in prevalence studies: modification of an existing tool and evidence of interrater agreement. J Clin Epidemiol. 2012;65(9). 934‐9. 10.1016/j.jclinepi.2011.11.014. 22742910

[20] Welton NJ , Sutton AJ , Cooper NJ , Abrams KR , Ades AE . Evidence Synthesis for Decision Making in Healthcare. Wiley; 2012.

[21] Resnik SS , Shelley WB . Penicillin hypersensitivity: detection by basophil response to challenge. J Invest Dermatol. 1965;45(4):269‐274. 5320053 10.1038/jid.1965.128

[22] Levine BB , Zolov DM . Prediction of penicillin allergy by immunological tests. J Allergy. 1969;43(4):231‐244. 10.1016/0021-8707(69)90066-5. 5251662

[23] Bierman CW , VanArsdel P, Jr . Penicillin allergy in children: the role of immunological tests in its diagnosis. J Allergy. 1969;43(5):267‐272. 10.1016/0021-8707(69)90147-6. 5253298

[24] Wide L , Juhlin L . Detection of penicillin allergy of the immediate type by radioimmunoassay of reagins (IgE) to penicilloyl conjugates. Clin Exp Allergy. 1971;1(2):171‐177. 10.1111/j.1365-2222.1971.tb03016.x. 4131681

[25] Warrington RJ , Simons FER , Ho HW , Gorski BA . Diagnosis of penicillin allergy by skin testing: the Manitoba experience. Can Med Assoc J. 1978;118(7):787‐791. 638909 PMC1818216

[26] Sullivan TJ , Wedner HJ , Shatz GS , Yecies LD , Parker CW . Skin testing to detect penicillin allergy. J Allergy Clin Immunol. 1981;68(3):171‐180. 10.1016/0091-6749(81)90180-9. 6267115

[27] VanArsdel PP, Jr , Martonick GJ , Johnson LE , Sprenger JD , Altman LC , Henderson WR, Jr . The value of skin testing for penicillin allergy diagnosis. West J Med. 1986;144(3):311‐314. 3962293 PMC1306602

[28] Graff‐Lonnevig V , Hedlin G , Lindfors A . Penicillin allergy—a rare paediatric condition? Arch Dis Child. 1988;63(11):1342‐1346. 10.1136/adc.63.11.1342. 2974274 PMC1779154

[29] Blanca M , Vega JM , Garcia J , et al. Allergy to penicillin with good tolerance to other penicillins; study of the incidence in subjects allergic to betalactams. Clin Exp Allergy. 1990;20(5):475‐481. 10.1111/j.1365-2222.1990.tb03139.x. 2253079

[30] Surtees SJ , Stockton MG , Gietzen TW . Allergy to penicillin: fable or fact? BMJ. 1991;302(6784):1051‐1052. 1903664 10.1136/bmj.302.6784.1051 PMC1669643

[31] Sogn DD , Evans R, III , Shepherd GM , et al. Results of the National Institute of allergy and infectious diseases Collaborative clinical trial to test the predictive value of skin testing with major and minor penicillin derivatives in hospitalized adults. Arch Intern Med. 1992;152(5):1025‐1032. 10.1001/archinte.1992.00400170105020. 1580706

[32] Gadde J , Spence M , Wheeler B , Adkinson NF . Clinical‐experience with penicillin skin testing in a large inner‐city STD Clinic. J Am Med Assoc. 1993;270(20):2456‐2463. 10.1001/jama.270.20.2456. 8230623

[33] Sastre J , Quijano LD , Novalbos A , et al. Clinical cross‐reactivity between amoxicillin and cephadroxil in patients allergic to amoxicillin and with good tolerance of penicillin. Allergy. 1996;51(6):383‐386. 8837660

[34] Macy E , Richter PK , Falkoff R , Zeiger R . Skin testing with penicilloate and penilloate prepared by an improved method: amoxicillin oral challenge in patients with negative skin test responses to penicillin reagents. J Allergy Clin Immunol. 1997;100(5):586‐591. 10.1016/s0091-6749(97)70159-3. 9389285

[35] Hervé M , De la Rocque F , Bouhanna A , Albengres E , Reinert P . Exploration of 112 children suspected of amoxicillin allergy. Indications and efficacy of oral provocation test. Arch Pediatr. 1998;5(5):503‐509. 10.1016/S0929-693X(99)80314-5. 9759183

[36] Pichichero ME , Pichichero DM . Diagnosis of penicillin, amoxicillin, and cephalosporin allergy: reliability of examination assessed by skin testing and oral challenge. J Pediatr. 1998;132(1):137‐143. 10.1016/S0022-3476(98)70499-8. 9470015

[37] Warrington RJ , Lee KR , McPhillips S . The value of skin testing for penicillin allergy in an inpatient population: analysis of the subsequent patient management. Allergy Asthma Proc. 2000;21(5):297‐299. 11061039 10.2500/108854100778248269

[38] Forrest DM , Schellenberg RR , Thien VVS , King S , Anis AH , Dodek PM . Introduction of a practice guideline for penicillin skin testing improves the appropriateness of antibiotic therapy. Clin Infect Dis. 2001;32(12):1685‐1690. 10.1086/320752. 11360207

[39] Lebel B , Messaad D , Kvedariene V , Rongier M , Bousquet J , Demoly P . Cysteinyl‐leukotriene release test (CAST) in the diagnosis of immediate drug reactions. Allergy. 2001;56(7):688‐692. 11421930 10.1034/j.1398-9995.2001.00103.x

[40] Langley JM , Halperin SA , Bortolussi R . History of penicillin allergy and referral for skin testing: evaluation of a pediatric penicillin allergy testing program. Clin Invest Med. 2002;25(5):181‐184. 12375719

[41] Solensky R , Earl HS , Gruchalla RS . Lack of penicillin resensitization in patients with a history of penicillin allergy after receiving repeated penicillin courses. Arch Intern Med. 2002;162(7):822‐826. 10.1001/archinte.162.7.822. 11926858

[42] Torres MJ , Mayorga C , Leyva L , et al. Controlled administration of penicillin to patients with a positive history but negative skin and specific serum IgE tests. Clin Exp Allergy. 2002;32(2):270‐276. 10.1046/j.1365-2222.2002.01296.x. 11929493

[43] Perrin F , Lavaud F , Rey JB , Gourdier B , Lebargy F . Drug allergy and challenge protocols: a three‐year follow‐up in Reims university hospital. J de Pharm Clinique. 2004;23(2):63‐68.

[44] Stember RH . Prevalence of skin test reactivity in patients with convincing, vague, and unacceptable histories of penicillin allergy. Allergy Asthma Proc. 2005;26(1):59‐64. 15813290

[45] Wong BBL , Keith PK , Waserman S . Clinical history as a predictor of penicillin skin test outcome. Ann Allergy Asthma Immunol. 2006;97(2):169‐174. 10.1016/s1081-1206(10)60008-7. 16937746

[46] del Real GA , Rose ME , Ramirez‐Atamoros MT , et al. Penicillin skin testing in patients with a history of beta‐lactam allergy. Ann Allergy Asthma Immunol. 2007;98(4):355‐359. 10.1016/S1081-1206(10)60882-4. 17458432

[47] Romano A , Viola M , Bousquet PJ , et al. A comparison of the performance of two penicillin reagent kits in the diagnosis of beta‐lactam hypersensitivity. Allergy. 2007;62(1):53‐58. 10.1111/j.1398-9995.2006.01272.x. 17156342

[48] Goldberg A , Confino‐Cohen R . Skin testing and oral penicillin challenge in patients with a history of remote penicillin allergy. Ann Allergy Asthma Immunol. 2008;100(1):37‐43. 10.1016/s1081-1206(10)60402-4. 18254480

[49] Nolan RC , Puy R , Deckert K , O'Hehir RE , Douglass JA . Experience with a new commercial skin testing kit to identify IgE‐mediated penicillin allergy. Intern Med J. 2008;38(5):357‐361. 10.1111/j.1445-5994.2008.01657.x. 18402562

[50] Rodriguez‐Alvarez M , Santos‐Magadan S , Rodriguez‐Jimenez B , de Arellano IRR , Vazquez‐Cortes S , Martinez‐Cocera C . Reproducibility of delayed‐type reactions to betalactams. Allergol Immunopathol. 2008;36(4):201‐214. 10.1016/s0301-0546(08)72550-3. 18928686

[51] Tantikul C , Dhana N , Jongjarearnprasert K , Visitsunthorn N , Vichyanond P , Jirapongsananuruk O . The utility of the World health Organization‐the uppsala Monitoring centre (WHO‐UMC) system for the assessment of adverse drug reactions in hospitalized children. Asian Pac J Allergy Immunol. 2008;26(2‐3):77‐82. 19054924

[52] Waton J , Trechot P , Loss‐Ayav C , Schmutz JL , Barbaud A . Negative predictive value of drug skin tests in investigating cutaneous adverse drug reactions. Br J Dermatol. 2009;160(4):786‐794. 10.1111/j.1365-2133.2008.08975.x. 19120340

[53] Macy E , Goldberg B , Poon KY . Use of commercial anti‐penicillin IgE fluorometric enzyme immunoassays to diagnose penicillin allergy. Ann Allergy Asthma Immunol. 2010;105(2):136‐141. 10.1016/j.anai.2010.06.014. 20674824

[54] Aun MV , Bisaccioni C , Garro LS , et al. Outcomes and safety of drug provocation tests. Allergy Asthma Proc. 2011;32(4):301‐306. 10.2500/aap.2011.32.3450. 21781406

[55] Borch JE , Bindslev‐Jensen C . Full‐course drug challenge test in the diagnosis of delayed allergic reactions to penicillin. Int Arch Allergy Immunol. 2011;155(3):271‐274. 10.1159/000320384. 21293146

[56] Caubet JC , Kaiser L , Lemaitre B , Fellay B , Gervaix A , Eigenmann PA . The role of penicillin in benign skin rashes in childhood: a prospective study based on drug rechallenge. J Allergy Clin Immunol. 2011;127(1):218‐222. 10.1016/j.jaci.2010.08.025. 21035175 PMC7126001

[57] Holm A , Mosbech H . Challenge test results in patients with suspected penicillin allergy, but No specific IgE. Allergy Asthma Immunol Res. 2011;3(2):118‐122. 10.4168/aair.2011.3.2.118. 21461251 PMC3062790

[58] Kamboj S , Yousef E , McGeady S , Hossain J . The prevalence of antibiotic skin test reactivity in a pediatric population. Allergy Asthma Proc. 2011;32(2):99‐105. 10.2500/aap.2011.32.3433. 21439162

[59] Moral L , Garde J , Toral T , Fuentes MJ , Marco N . Short protocol for the study of paediatric patients with suspected betalactam antibiotic hypersensitivity and low risk criteria. Allergol Immunopathol. 2011;39(6):337‐341. 10.1016/j.aller.2010.10.002. 21429652

[60] Na HR , Lee JM , Jung JW , Lee SY . Usefulness of drug provocation tests in children with a history of adverse drug reaction. Korean J Pediatr. 2011;54(7):304‐309. 10.3345/kjp.2011.54.7.304. 22025924 PMC3195796

[61] Ponvert C , Perrin Y , Bados‐Albiero A , et al. Allergy to betalactam antibiotics in children: results of a 20‐year study based on clinical history, skin and challenge tests. Pediatr Allergy Immunol. 2011;22(4):411‐418. 10.1111/j.1399-3038.2011.01169.x. 21535179

[62] Richter AG , Wong G , Goddard S , et al. Retrospective case series analysis of penicillin allergy testing in a UK specialist regional allergy clinic. J Clin Pathol. 2011;64(11):1014‐1018. 10.1136/jcp.2010.088203. 21742749

[63] Seitz CS , Brocker EB , Trautmann A . Diagnosis of drug hypersensitivity in children and adolescents: discrepancy between physician‐based assessment and results of testing. Pediatr Allergy Immunol. 2011;22(4):405‐410. 10.1111/j.1399-3038.2011.01134.x. 21309856

[64] Garcia Nunez I , Barasona Villarejo MJ , Algaba Marmol MA , Moreno Aguilar C , Guerra Pasadas F . Diagnosis of patients with immediate hypersensitivity to beta‐lactams using retest. J Investig Allergol Clin Immunol. 2012;22(1):41‐47. 22448453

[65] Hjortlund J , Mortz CG , Skov PS , et al. One‐week oral challenge with penicillin in diagnosis of penicillin allergy. Acta Derma Venereol. 2012;92(3):307‐312. 10.2340/00015555-1254. 22170236

[66] Kopac P , Zidarn M , Kosnik M . Epidemiology of hypersensitivity reactions to penicillin in Slovenia. Acta Dermatovenerol Alp Pannonica Adriat. 2012;21(4):65‐67. 23599125

[67] Erkocoglu M , Kaya A , Civelek E , et al. Prevalence of confirmed immediate type drug hypersensitivity reactions among school children. Pediatr Allergy Immunol. 2013;24(2):160‐167. 10.1111/pai.12047. 23373964

[68] Hjortlund J , Mortz CG , Skov PS , Bindslev‐Jensen C . Diagnosis of penicillin allergy revisited: the value of case history, skin testing, specific IgE and prolonged challenge. Allergy. 2013;68(8):1057‐1064. 10.1111/all.12195. 23889703

[69] Kao L , Rajan J , Roy L , Kavosh E , Khan DA . Adverse reactions during drug challenges: a single US institution's experience. Ann Allergy Asthma Immunol. 2013;110(2):86‐91. 10.1016/j.anai.2012.11.007. 23352526

[70] Macy E , Ngor EW . Safely diagnosing clinically significant penicillin allergy using only penicilloyl‐poly‐lysine, penicillin, and oral amoxicillin. J Allergy Clin Immunol Pract. 2013;1(3):258‐263. 10.1016/j.jaip.2013.02.002. 24565482

[71] Rimawi RH , Cook PP , Gooch M , et al. The impact of penicillin skin testing on clinical practice and antimicrobial stewardship. J Hosp Med. 2013;8(6): 341‐345. 10.1002/jhm.2036. 23553999

[72] Al‐Ahmad M , Rodriguez Bouza T , Arifhodzic N . Penicillin allergy evaluation: experience from a drug allergy clinic in an Arabian Gulf Country, Kuwait. Asia Pac Allergy. 2014;4(2):106‐112. 10.5415/apallergy.2014.4.2.106. 24809016 PMC4005349

[73] Colas H , David V , Molle I , Bernier C , Magnan A , Pipet A . Drug provocation tests for children: in‐hospital or in an outpatient consultation? A protocol comparing the two possibilities. Rev Francaise D Allergol. 2014;54(4):300‐316. 10.1016/j.reval.2014.01.038.

[74] Fox SJ , Park MA . Penicillin skin testing is a safe and effective tool for evaluating penicillin allergy in the pediatric population. J Allergy Clin Immunol Pract. 2014;2(4):439‐444. 10.1016/j.jaip.2014.04.013. 25017533

[75] Picard M , Paradis L , Bégin P , Paradis J , Des Roches A . Skin testing only with penicillin G in children with a history of penicillin allergy. Ann Allergy Asthma Immunol. 2014;113(1):75‐81. 10.1016/j.anai.2014.04.017. 24856884

[76] Trautmann A , Seitz CS , Stoevesandt J , Kerstan A . Aminopenicillin‐associated exanthem: lymphocyte transformation testing revisited. Clin Exp Allergy. 2014;44(12):1531‐1538. 10.1111/cea.12437. 25323308

[77] Zambonino MA , Corzo JL , Munoz C , et al. Diagnostic evaluation of hypersensitivity reactions to beta‐lactam antibiotics in a large population of children. Pediat Allergy Immunol. 2014;25(1):80‐87. 10.1111/pai.12155. 24329898

[78] Barni S , Mori F , Sarti L , et al. Utility of skin testing in children with a history of non‐immediate reactions to amoxicillin. Clin Exp Allergy. 2015;45(9):1472‐1474. 10.1111/cea.12596. 26178175

[79] Ben‐Said B , Arnaud‐Butel S , Rozieres A , et al. Allergic delayed drug hypersensitivity is more frequently diagnosed in drug reaction, eosinophilia and systemic symptoms (DRESS) syndrome than in exanthema induced by beta‐lactam antibiotics. J Dermatol Sci. 2015;80(1):71‐74. 10.1016/j.jdermsci.2015.07.014. 26341696

[80] Blanca‐Lopez N , Perez‐Alzate D , Ruano F , et al. Selective immediate responders to amoxicillin and clavulanic acid tolerate penicillin derivative administration after confirming the diagnosis. Allergy. 2015;70(8):1013‐1019. 10.1111/all.12636. 25913298

[81] Bourke J , Pavlos R , James I , Phillips E . Improving the effectiveness of penicillin allergy de‐labeling. J Allergy Clin Immunol Pract. 2015;3(3):365‐374. 10.1016/j.jaip.2014.11.002. 25609352

[82] Murphy K , Scanlan B , Coghlan D . Does this child really have a penicillin allergy? Ir Med J. 2015;108(4):103. 26016298

[83] Paukert J , Kopelentová E , Dvořáková L , et al. Allergy to beta‐lactam antibiotics in children. Cesko‐Slovenska Pediatr. 2015;70(1):9‐13.

[84] Rosenfield L , Kalicinsky C , Warrington R . A retrospective comparison of false negative skin test rates in penicillin allergy, using pencilloyl‐poly‐lysine and minor determinants or Penicillin G, followed by open challenge. Allergy Asthma Clin Immunol. 2015;11:34. 10.1186/s13223-015-0098-5. 26594228 PMC4654886

[85] Abrams EM , Wakeman A , Gerstner TV , Warrington RJ , Singer AG . Prevalence of beta‐lactam allergy: a retrospective chart review of drug allergy assessment in a predominantly pediatric population. Allergy Asthma Clin Immunol. 2016;12(12):59–64. 10.1186/s13223-016-0165-6. 27956906 PMC5129666

[86] Atanaskovic‐Markovic M , Gaeta F , Medjo B , et al. Non‐immediate hypersensitivity reactions to beta‐lactam antibiotics in children—our 10‐year experience in allergy work‐up. Pediatr Allergy Immunol. 2016;27(5):533‐538. 10.1111/pai.12565. 26999792 PMC7167905

[87] Choi J , Lee JY , Kim KH , Choi J , Ahn K , Kim J . Evaluation of drug provocation tests in Korean children: a single center experience. Asia Pac J Allergy Immunol. 2016;34(2):130‐136. 10.12932/AP0692.34.2.2016. 27007834

[88] King EA , Challa S , Curtin P , Bielory L . Penicillin skin testing in hospitalized patients with beta‐lactam allergies: effect on antibiotic selection and cost. Ann Allergy Asthma Immunol. 2016;117(1):67‐71. 10.1016/j.anai.2016.04.021. 27211057

[89] Manuyakorn W , Singvijarn P , Benjaponpitak S , et al. Skin testing with β‐lactam antibiotics for diagnosis of β‐lactam hypersensitivity in children. Asia Pac J Allergy Immunol. 2016;34(3):242‐247. 10.12932/AP0750. 27543729

[90] Mill C , Primeau MN , Medoff E , et al. Assessing the diagnostic properties of a graded oral provocation challenge for the diagnosis of immediate and nonimmediate reactions to amoxicillin in children. JAMA Pediatr. 2016;170(6):e160033. 10.1001/jamapediatrics.2016.0033. 27043788

[91] Moreno E , Laffond E , Munoz‐Bellido F , et al. Performance in real life of the European Network on Drug Allergy algorithm in immediate reactions to beta‐lactam antibiotics. Allergy. 2016;71(12):1787‐1790. 10.1111/all.13032. 27543745

[92] Mota I , Gaspar A , Chambel M , Piedade S , Morais‐Almeida M . Hypersensitivity to beta‐lactam antibiotics: a three‐year study. Eur Ann Allergy Clin Immunol. 2016;48(6):212‐219. 27852424

[93] Ratzon R , Reshef A , Efrati O , et al. Impact of an extended challenge on the effectiveness of β‐lactam hypersensitivity investigation. Ann Allergy Asthma Immunol. 2016;116(4):329‐333. 10.1016/j.anai.2016.01.018. 26922211

[94] Specjalski K , Kita‐Milczarska K , Chelminska M , Jassem E . Typing safe antibiotics in amoxicillin hypersensitive patients—development of a stepwise protocol. Pneumonol Alergol Pol. 2016;84(1):16‐21. 10.5603/PiAP.2016.0001. 26806417

[95] Vezir E , Misirlioglu ED , Civelek E , et al. Direct oral provocation tests in non‐immediate mild cutaneous reactions related to beta‐lactam antibiotics. Pediat Allergy Immunol. 2016;27(1):50‐54. 10.1111/pai.12493. 26619970

[96] Chen JR , Tarver SA , Alvarez KS , Tran T , Khan DA . A proactive approach to penicillin allergy testing in hospitalized patients. J Allergy Clin Immunol Pract. 2017;5(3):686‐693. 10.1016/j.jaip.2016.09.045. 27888034

[97] Chiriac AM , Rerkpattanapipat T , Bousquet PJ , Molinari N , Demoly P . Optimal step doses for drug provocation tests to prove beta‐lactam hypersensitivity. Allergy. 2017;72(4):552‐561. 10.1111/all.13037. 27569064

[98] Confino‐Cohen R , Rosman Y , Meir‐Shafrir K , et al. Oral challenge without skin testing safely excludes clinically significant delayed‐onset penicillin hypersensitivity. J Allergy Clin Immunol Pract. 2017;5(3):669‐675. 10.1016/j.jaip.2017.02.023. 28483317

[99] Iammatteo M , Ferastraoaru D , Koransky R , et al. Identifying allergic drug reactions through placebo‐controlled graded challenges. J Allergy Clin Immunol Pract. 2017;5(3):711‐717. 10.1016/j.jaip.2016.09.041. 27888028

[100] Lezmi G , Alrowaishdi F , Bados‐Albiero A , Scheinmann P , de Blic J , Ponvert C . Non‐immediate‐reading skin tests and prolonged challenges in non‐immediate hypersensitivity to beta‐lactams in children. Pediat Allergy Immunol. 2018;29(1):84‐89. 10.1111/pai.12826. 29047169

[101] Marwood J , Aguirrebarrena G , Kerr S , Welch SA , Rimmer J . De‐labelling self‐reported penicillin allergy within the emergency department through the use of skin tests and oral drug provocation testing. Emerg Med Australas. 2017;29(5):509‐515. 10.1111/1742-6723.12774. 28378949

[102] Mawhirt SL , Fonacier LS , Calixte R , Davis‐Lorton M , Aquino MR . Skin testing and drug challenge outcomes in antibiotic‐allergic patients with immediate‐type hypersensitivity. Ann Allergy Asthma Immunol. 2017;118(1):73‐79. 10.1016/j.anai.2016.10.003. 27864093

[103] Misirlioglu ED , Guvenir H , Toyran M , et al. Frequency of selective immediate responders to aminopenicillins and cephalosporins in Turkish children. Allergy Asthma Proc. 2017;38(5):376‐382. 10.2500/aap.2017.38.4065. 28814358

[104] Sundquist BK , Bowen BJ , Otabor U , Celestin J , Sorum PC . Proactive penicillin allergy testing in primary care patients labeled as allergic: outcomes and barriers. Postgrad Med. 2017;129(8):915‐920. 10.1080/00325481.2017.1370360. 28829234

[105] Tucker MH , Lomas CM , Ramchandar N , Waldram JD . Amoxicillin challenge without penicillin skin testing in evaluation of penicillin allergy in a cohort of Marine recruits. J Allergy Clin Immunol Pract. 2017;5(3):813‐815. 10.1016/j.jaip.2017.01.023. 28341170

[106] Vyles D , Adams J , Chiu A , Simpson P , Nimmer M , Brousseau DC . Allergy testing in children with low‐risk penicillin allergy symptoms. Pediatrics. 2017;140(2):e20170471. 10.1542/peds.2017-0471. 28674112

[107] Anterasian CM , Geng B . Penicillin skin testing in the management of penicillin allergy in an outpatient pediatric population. Allergy Asthma Proc. 2018;39(4):305‐310. 10.2500/aap.2018.39.4138. 30095396 PMC9292462

[108] Faitelson Y , Boaz M , Dalal I . Asthma, family history of drug allergy, and age predict amoxicillin allergy in children. J Allergy Clin Immunol Pract. 2018;6(4):1363‐1367. 10.1016/j.jaip.2017.11.015. 29226807

[109] Ibanez MD , Del Rio PR , Lasa EM , et al. Prospective assessment of diagnostic tests for pediatric penicillin allergy, from clinical history to challenge tests. Ann Allergy Asthma Immunol. 2018;121(2):235–244. 10.1016/j.anai.2018.05.013. 29803713

[110] Labrosse R , Paradis L , Lacombe‐Barrios J , et al. Efficacy and safety of 5‐day challenge for the evaluation of nonsevere amoxicillin allergy in children. J Allergy Clin Immunol Pract. 2018;6(5):1673‐1680. 10.1016/j.jaip.2018.01.030. 29425903

[111] Lindsey D , Banks T . Efficient identification and clearance of low‐risk penicillin allergy patients. Ann Allergy Asthma Immunol. 2018;121(5):S15. 10.1016/j.anai.2018.09.046.

[112] Macy E , Vyles D . Who needs penicillin allergy testing? Ann Allergy Asthma Immunol. 2018;121(5):523‐529. 10.1016/j.anai.2018.07.041. 30092265

[113] Noh SR , Yoon J , Cho HJ , et al. Outcomes of drug provocation tests in Korean children with suspected drug hypersensitivity reaction. Allergy Asthma Resp Dis. 2018;6(1):26‐33. 10.4168/aard.2018.6.1.26.

[114] Ramsey A , Staicu ML . Use of a penicillin allergy screening algorithm and penicillin skin testing for transitioning hospitalized patients to first‐line antibiotic therapy. J Allergy Clin Immunol Pract. 2018;6(4):1349‐1355. 10.1016/j.jaip.2017.11.012. 29242142

[115] Arnold A , Sommerfield A , Ramgolam A , et al. The role of skin testing and extended antibiotic courses in assessment of children with penicillin allergy: an Australian experience. J Paediatr Child Health. 2019;55(4):428‐432. 10.1111/jpc.14220. 30209846

[116] Blumenthal KG , Li Y , Hsu JT , et al. Outcomes from an inpatient beta‐lactam allergy guideline across a large US health system. Infect Control Hosp Epidemiol. 2019;40(5):528‐535. 10.1017/ice.2019.50. 30915929 PMC6536839

[117] Devchand M , Kirkpatrick CMJ , Stevenson W , et al. Evaluation of a pharmacist‐led penicillin allergy de‐labelling ward round: a novel antimicrobial stewardship intervention. J Antimicrob Chemother. 2019;74(6):1725‐1730. 10.1093/jac/dkz082. 30869124

[118] Diaferio L , Chiriac AM , Leoni MC , et al. Skin tests are important in children with beta‐lactam hypersensitivity, but may be reduced in number. Pediatr Allergy Immunol. 2019;30(4):462‐468. 10.1111/pai.13041. 30734416

[119] du Plessis T , Walls G , Jordan A , Holland DJ . Implementation of a pharmacist‐led penicillin allergy de‐labelling service in a public hospital. J Antimicrob Chemother. 2019;74(5):1438–1446. 10.1093/jac/dky575. 30753497

[120] Francelino EV , Arujo SR , Ferreira JFS , et al. Investigating true beta‐lactam allergy in the outpatient allergy clinics at a public Children's hospital, Ceara, Brazil. J Young Pharm. 2019;11(1):88‐91. 10.5530/jyp.2019.11.18.

[121] Rodriguez RG , Lozano LM , Ortega AE , Segade JB , Bonilla PG , Torrijos EG . Provocation tests in nonimmediate hypersensitivity reactions to beta‐lactam antibiotics in children: are extended challenges needed? J Allergy Clin Immunol Pract. 2019;7(1):265–269. 10.1016/j.jaip.2018.06.023. 30009988

[122] Iammatteo M , Alvarez Arango S , Ferastraoaru D , et al. Safety and outcomes of oral graded challenges to amoxicillin without prior skin testing. J Allergy Clin Immunol Pract. 2019;7(1):236‐243. 10.1016/j.jaip.2018.05.008. 29802906

[123] Jones BM , Avramovski N , Concepcion AM , Crosby J , Bland CM . Clinical and economic outcomes of penicillin skin testing as an antimicrobial stewardship Initiative in a Community health system. Open Forum Infect Dis. 2019;6(4):ofz109. 10.1093/ofid/ofz109. 30968057 PMC6451650

[124] Kennard L , Rutkowski K , Siew LQC , et al. Flucloxacillin hypersensitivity: patient outcomes in a multicenter retrospective study. J Allergy Clin Immunol Pract. 2019;7(7):2212–2217. 10.1016/j.jaip.2019.03.018. 30922988

[125] Kuruvilla M , Shih J , Patel K , Scanlon N . Direct oral amoxicillin challenge without preliminary skin testing in adult patients with allergy and at low risk with reported penicillin allergy. Allergy Asthma Proc. 2019;40(1):57‐61. 10.2500/aap.2019.40.4184. 30582497

[126] Mustafa SS , Conn K , Ramsey A . Comparing direct challenge to penicillin skin testing for the outpatient evaluation of penicillin allergy: a randomized, controlled trial. J Allergy Clin Immunol Pract. 2019;7(7):2163–2170. 10.1016/j.jaip.2019.05.037. 31170542

[127] Ramsey A , Holly AM , Mustafa SS , Staicu ML . Use of a penicillin allergy screening algorithm Incorporating direct challenges to manage penicillin allergic inpatients. J Allergy Clin Immunol. 2019;143(2), AB27.

[128] Savic L , Gurr L , Kaura V , et al. Penicillin allergy de‐labelling ahead of elective surgery: feasibility and barriers. Br J Anaesth. 2019;123(1):e110‐e116. 10.1016/j.bja.2018.09.009. 30915983

[129] Siew LQC , Li PH , Watts TJ , et al. Identifying low‐risk beta‐lactam allergy patients in a UK Tertiary centre. J Allergy Clin Immunol Pract. 2019;7(7):2173–2181. 10.1016/j.jaip.2019.03.015. 30922992

[130] Simsek IE , Cogurlu MT , Aydogan M . Suspected reaction with cephalosporin may Be a predictive factor for beta‐lactam allergy in children. Int Arch Allergy Immunol. 2019;178(3):248‐254. 10.1159/000494506. 30517941

[131] Solensky R , Jacobs J , Lester M , et al. Penicillin allergy evaluation: a prospective, multicenter, open‐label evaluation of a comprehensive penicillin skin test kit. J Allergy Clin Immunol Pract. 2019;7(6):1876–1885. 10.1016/j.jaip.2019.02.040. 30878711

[132] Vila L , Garcia V , Martinez Azcona O , Pineiro L , Meijide A , Balboa V . Mild to moderate hypersensitivity reactions to beta‐lactams in children: a single‐centre retrospective review. BMJ Paediatr Open. 2019;3(1):e000435. 10.1136/bmjpo-2019-000435. PMC6542429 31206079

[133] Copetti M , Copetti M , Fontana A , et al. Advances in meta‐analysis: examples from internal medicine to neurology. Neuroepidemiology. 2014;42(1):59‐67. 10.1159/000355433. 24356064

[134] Efthimiou O . Practical guide to the meta‐analysis of rare events. Evid Based Ment Health. 2018;21(2), 72‐76. 10.1136/eb-2018-102911. 29650528 PMC10270432

[135] Sompornrattanaphan M , Wongsa C , Kreetapirom P , Taweechue AJ , Theankeaw O , Thongngarm T . Fatal anaphylaxis from a second amoxicillin/clavulanic acid provocation after a prior negative provocation. J Allergy Clin Immunol Pract. 2020;8(2), 752‐754. 10.1016/j.jaip.2019.07.022. 31374359

[136] Blumenthal KG , Huebner EM , Fu X , et al. Risk‐based pathway for outpatient penicillin allergy evaluations. J Allergy Clin Immunol Pract. 2019;7(7). 2411‐2414. 10.1016/j.jaip.2019.04.006. 30981747 PMC6733651

[137] Sousa‐Pinto B , Tarrio I , Blumenthal KG , et al. Accuracy of penicillin allergy diagnostic tests: a systematic review and meta‐analysis. J Allergy Clin Immunol. 2021;147(1):296–308. 10.1016/j.jaci.2020.04.058. 32446963 PMC8019189

[138] Romano A , Atanaskovic‐Markovic M , Barbaud A , et al. Towards a more precise diagnosis of hypersensitivity to beta‐lactams—an EAACI position paper. Allergy. 2020;75(6):1300–1315. 10.1111/all.14122. 31749148

